# Age-dependent variations in proteomic characteristics of spermatozoa in Simmental bull

**DOI:** 10.3389/fvets.2024.1393706

**Published:** 2024-08-08

**Authors:** Faisal Amri Satrio, Ni Wayan Kurniani Karja, Mohamad Agus Setiadi, Ekayanti Mulyawati Kaiin, Berlin Pandapotan Pardede, Bambang Purwantara

**Affiliations:** ^1^Veterinary Medicine Study Program, Faculty of Medicine, Padjadjaran University, West Java, Bandung, Indonesia; ^2^Division of Reproduction and Obstetrics, School of Veterinary Medicine and Biomedical Sciences, IPB University, West Java, Bogor, Indonesia; ^3^Research Center for Applied Zoology, National Research and Innovation Agency (BRIN), West Java, Bogor, Indonesia

**Keywords:** age, bull, LC-MS/MS, post-thawing sperm, proteomic

## Abstract

Increasing the age of bulls results in a decrease in reproductive function, including a reduction in sperm quality, which plays a vital role in determining the fertility of bulls. Through a proteomic approach, this research aims to analyze the influence of age factors on various proteomes contained in bull sperm. Frozen semen samples from Simmental Bulls were categorized into three age groups: two, four, and ≥10 years old. Subsequently, the post-thaw sperm cells obtained were separated based on molecular weight using 1D-SDS-PAGE. Peptides extracted from the bands produced in each age group were subjected to LC-MS/MS analysis. A total of 72 protein types were identified, with 45 being detected in the 4-year-old group and 41 expressed in both the 2 and ≥10-year-old groups. The results provided insights into proteins' role in sperm metabolism across all age groups. Specifically, the 2-year-old group exhibited the expression of proteins associated with acrosome assembly and spermatid development (SPACA1). In contrast, those in the 4-year-old group were linked to motility (PEBP4) and sperm decapacitation factor (PEBP1). Proteins expressed in the 2 and -year-old groups were discovered to be involved in fertilization processes (TEX101). In contrast, the ≥10-year-old age group was associated with hyperactive movement related to capacitation (Tubulin). In conclusion, age influenced the differences observed in the proteomic profile of post-thaw Simmental bull sperm using the 1D-SDS-PAGE tandem LC-MS/MS approach.

## Introduction

Bull fertility is defined as the ability of sperm to fertilize and activate oocytes and support embryo development (1). This factor plays a crucial role in reproductive efficiency and the success of artificial insemination (AI) in bulls. The age of the bull is widely believed to have an impact on fertility. Previous studies categorized bull age as young (1.8-2 years) and adult (3-6 years) (2–4). Satrio et al. (5) showed that optimal semen production in bulls was achieved between 3 and 4 years of age. Collins et al. (6) discovered the highest fertility rates in Holstein and Guernsey bulls at 3-4 years of age. Generally, the reproductive capacity of bulls gradually decreases after reaching the highest fertility age due to age-related declines in various reproductive functions, such as histological (7, 8) and clinical (9) deterioration in testicular function. Changes in testicular function can disrupt spermatogenesis (8) and reduce semen quality (2, 5, 10–12). Despite this, old bulls (over 10 years old) are still maintained and used for frozen semen production in AI activities at the Indonesian Artificial Insemination Center.

Several methods have been employed to predict bull fertility, including evaluating non-return rates (NRR) (13). Additionally, conventional assessments of semen quality, such as plasma membrane integrity, motility, morphology, and acrosome, are routinely performed to predict fertility (1, 14). In Indonesia, the SNI 4869-2:2017 standard for frozen semen quality in bulls includes motility, abnormalities, individual movement, sperm concentration, and recovery rate percentage to achieve good fertility. These standard semen evaluation procedures are visually helpful in determining poor-quality semen but insufficient in accurately detecting potential markers of bull fertility (1, 14). Therefore, sperm quality must be evaluated through a molecular approach to obtain important information concerning the potential markers.

Proteomics has become the most advanced approach for predicting fertility with increased accuracy (15). By employing this method, the molecular aspects of sperm that impact fertility can be identified (16–18). Sperm proteomic analysis is used to determine the essential functions of proteins and their regulatory roles in various fertilization processes (16, 19, 20). Furthermore, alterations in proteomic expression are believed to play a crucial role in the transition of sperm function from the epididymis to capacitation in the female reproductive tract and subsequent fertilization (16, 17). Previous studies discovered fertility markers in bull semen (13, 16, 19, 21, 22). Only limited information exists on the proteomic analysis of post-thaw sperm about bull age. Therefore, this study aims to investigate the variations in the proteomic profile of post-thaw sperm among bulls of different ages.

## Materials and methods

### Experimental design and semen samples

This study only used frozen semen, a commercial product from an AI center, as the primary sample and did not directly involve bulls. Furthermore, the entire semen collection process was carried out using an artificial vagina for the same period (without seasonal differences), frozen using the same extender. To eliminate any potential for variation in the samples, the bulls were kept in the same environment regarding feeding and handling management. However, each stage follows the operational standards in Indonesia, SNI ISO 9001:2015 No. 824 100 16072, supervised by a veterinarian, considered the principles of animal welfare, which refer to the ethical clearance requirements of the Animal Care and Uses Committee. The AI Center owned all bulls used in this study under the auspices of the government. However, we only use commercial products sold to the public without requiring animal ownership approval. A total of 27 frozen semen straws (nine straws per each group) of nine Simmental bulls of different age groups, namely two (young; *n* = 3 bulls), four (adult; *n* = 3 bulls), and ≥10 years old (old; *n* = 3 bulls). The number of bulls used in this study is the total number of Simental bulls available at the AI Center during the research period.

### Extraction of post-thaw sperm proteins

The frozen semen was thawed at 37°C and washed thrice with phosphate-buffered saline through centrifugation at 1800 rpm for 10 min. The sperm pellet was then subjected to extraction using PRO-PREP™ Protein extraction solution (iNtRON Biotechnology, Korea) according to the manufacturer's instructions. 500 μL of PRO-PREP™ solution was added to the pellet, incubated at −20°C for 20 min, and centrifuged at 13000 rpm (4°C) for 5 min. The total soluble protein concentration of the sample was determined before SDS-PAGE analysis using the Bradford method (23), with BSA (Sigma-Aldrich) serving as the standard.

### Separation of sperm protein using SDS-PAGE

Protein separation was performed using a 12.5% polyacrylamide gel containing sodium dodecyl sulfate (SDS) and a 4% stacking gel. This process was carried out at a voltage of 60 V and a current of 20 mA for 3.5 h. Subsequently, the gel was stained using Coomassie Brilliant Blue staining (24). The marker employed was Excelband™ 3-color Broad Range Protein Marker PM2700 (SMOBIO^®^ Technology, Inc., Taiwan) with a molecular weight range of ~5-245 kDa.

### Liquid chromatography-mass spectrometry (LC-MS/MS) analysis

The protein bands formed on the gel were excised and washed twice with 200 μL of destaining solution [80 mg ammonium bicarbonate in 20 mL acetonitrile (ACN) and 20 mL ultrapure water] for 30 min at 37°C. Before digestion, the protein samples were treated with 30 μL of alkylation buffer [Iodoacetamide (IAA)] for 1 h at room temperature in the dark. Tryptic digestion was performed using 10 ng/μL of activated trypsin (Promega, Fitchburg, WI, USA), with an enzyme/substrate ratio of 1/50 (*w/w*) for 4 h at 37°C. A total 1% of the final volume of TCA (trichloroacetic acid) solution was added to stop the trypsin activity reaction (25). Furthermore, the activated peptide samples were purified with C18 Spin Columns (Thermo Scientific, Pierce Biotechnology, N Meridian Rd, Rockford, IL, USA), each containing 8 mg of C18 reversed-phase resin (to bind the peptides).

The dried peptide samples were dissolved in 50 μL of dissolving solution (2% ACN, 98% ultrapure water, and 0.1% formic acid) and centrifuged at 12000 rpm for 10 min. Subsequently, 2.5 μL of the peptides were fractionated using the Nano LC Ultimate 3000 Series System coupled with the Q Exactive™ Plus Hybrid Quadrupole-Orbitrap™ Mass Spectrometer (Thermo Fisher Scientific, Bremen, Germany). The trap column used had a diameter of 30 μm and a length of 5 mm (Thermo Scientific™ 164649, Bremen, Germany). The capillary column was the PepMap RSLC C18 column (75 μm inner diameter X 15 cm, 3 μm particle size, 100 pore size, part number ES 800) (Thermo Scientific, Bremen, Germany) with a flow rate of 300 nL/min. The eluents applied were H_2_O+0.1% formic acid (A) and 98% acetonitrile + 0.1% formic acid (B). The procedure for elution of peptides on a column includes 0–3 min gradient of solvent B; 2–35% solvent B for 3–30 min; 35–90% solvent B for 30–45 min; 90% solvent B for 45–90 min; and 5% solvent B for 60–90 min. The signal peptide was obtained using the LTQ-Orbitrap mass spectrometer (Thermo Scientific, Bremen, Germany) with a 200-2000 m/z mass range. The scans were acquired via 30, 000 MS resolution (at m/z 400) in the Orbitrap analyzer, followed by 10 intensive MS/MS scans of the precursor via collision-induced dissociation (CID) fragmentation at normalized collision energies of 35% (26).

### Protein identification

The data collected from the LC-MS/MS instrument were analyzed using Proteome Discoverer 2.2 software (Thermo Fisher Scientific) with the Sequest HT search engine, Uniprot bovine (Bos taurus) protein database (https://www.uniprot.org/). Proteins were required to have a sequence HT score > 0 and a minimum of two unique peptides, with a mass tolerance of 10 ppm. Those originating from contaminants such as keratin, egg yolk extender, and skim milk were excluded from the analysis. Moreover, the identified proteins were subjected to functional analysis using the online PANTHER classification system (pantherdb.org). Venn analysis, representing the intersection of each group, was conducted using Venny 2.1.0 https://bioinfogp.cnb.csic.es/tools/venny/. Protein interactions were analyzed with the STRING version 12.0 (https://string-db.org/) (27).

## Results

### Protein distribution and venn analysis

**Figure 1:**
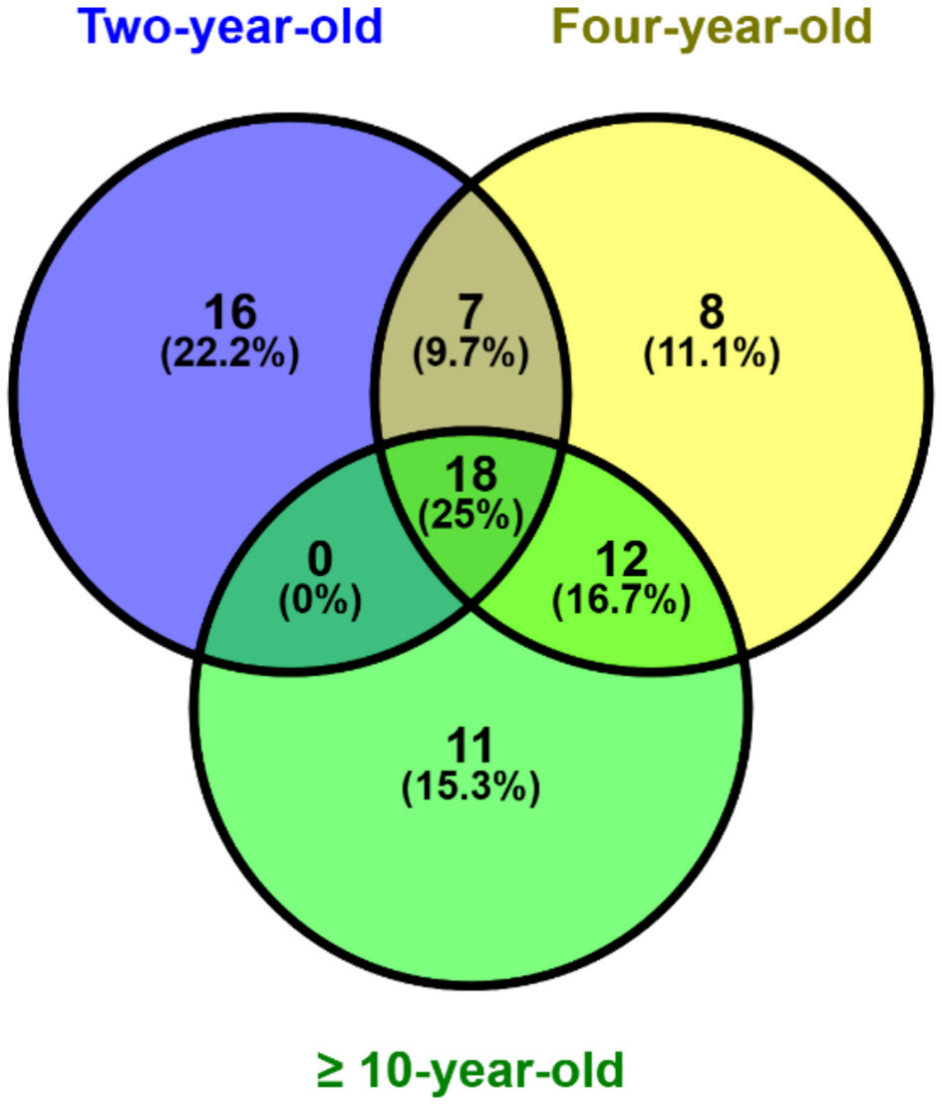
Post-thaw sperm protein expression at different ages bull by venn diagram analysis.

The results of the analysis showed that 41 sperm proteins were found in the 2-year age group, 45 sperm proteins in the 4-year age group, and 41 sperm proteins were found in the age group of more than 10 years. The analysis conducted using the Venny software (Figure 1) revealed that 18 proteins (25%) were expressed in all age groups, while 16 (22.2%), 8 (11.1%), and 11 (15.3%) were respectively expressed in each group, and the remaining were present in overlapping age groups.

### Identification of the expressed proteins and their function

The expressed proteins related to fertility and age are presented in Table 1. Across all age groups, proteins associated with essential sperm functions such as sperm metabolism, capacitation, acrosome reaction, and fertilization were expressed. In the 2-year-old group, there were proteins related to acrosome assembly (SPACA1), capacitation (LPL, BSP5, SRN), spermatogenesis (RNASE4), fertilization (TIMP2, ARSA, SPADH1, and SPADH2), antioxidant (PRDX5), and apoptosis (CYCT). The 4-year-old group had proteins related to decapacitation (PEBP1), antioxidants (QSOX1), capacitation (GSN and APOA4), and fertilization (C4BPA). The ≥10-year-old groups expressed proteins linked to cytoskeletal integrity (α- and β-tubulin). The 2 and 4-year-old groups contained proteins related to sexual reproduction (TEX101), motility (PRKAR1A), and fertilization (SERPINA5, SERPINE2), while those found in the four and ≥10-year-old groups were associated with sexual reproduction (DLD, ATP1A4, ADM1B, ADAM20).

**Table 1:** Post-thaw sperm protein expression at different ages of bulls is related to reproductive function.

**Accession no**.	**Protein name**	**Gen name**	**Biological process/molecular function/cellular component**
**All age groups**			
Q32KN6	Phosphoglycerate kinase 2	PGK2	Glycolysis Process, ATP-binding, tricarboxylic acid cycle,
P20004	Aconitate 2	ACO2	Tricarboxylic acid cycle, iron-ion binding, mitochondrial matrix/cytosol
Q29RK1	Citrate synthase	CS	Tricarboxylic acid cycle, citrate (Si)-synthase activity, mitochondrial matrix
Q32LG3	Malate dehydrogenase 2	MDH2	Aerobic respiration, L-malate dehydrogenase activity, mitochondrial matrix
E1B7S8	Acrosin binding protein	ACRBP	Fertilization/acrosome assembly, acrosomal vesicle
F1MQJ0	Angiotensin I converting enzyme	ACE	Male gonad development/spermatogenesis, metallopeptidase activity, plasma membrane (external side)
F1MRD0	Actin, cytoplasmic 1	ACTB	Cytoskeleton integrity
F1MTV1	Sperm adhesion molecule 1 (PH-20 hyaluronidase, zona pellucida binding)	SPAM1	Sperm fusion to membrane plasm of oocyte, hyaluronoglucosaminidase activity, acrosomal vesicle/cytoplasmic vesicle/plasma membrane
F1N2F2	Phosphoglycerate mutase 2	PGAM2	Glycolysis Process, phosphoglycerate mutase activity
Q3ZBD7	Glucose-6-phosphate isomerase	GPI	Converts glucose-6-phosphate to fructose-6-phosphate; important for glycolysis and ATP yield for sperm motility; prevents apoptosis and oxidative stress-induced cellular events
A6QPE2	IZUMO family member 4	IZUMO4	Reaction and sperm-oocyte/membrane binding/acrosome
F1N369	Zona pellucida binding protein	ZPBP	*Binding* sperm to zona pellucida/acrosomal assembly, receptor of zona pellucida complex/ acrosomal vesicle
F1MB08	Alpha-enolase	ENO1	Positive regulation of ATP biosynthetic process, GTPase binding, plasma membrane/cell surface
P79343	Acrosin	Bovine proacrosine	Single fertilization/ acrosome reaction, copper-ion binding, acrosomal matrix
**2-year-old group**			
F1MN30	Sperm acrosome membrane-associated protein 1	SPACA1	Acrosomal assembly, acrosomal membrane; spermatid development; acrosomal vesicle, plasma membrane
P11151	Lipoprotein lipase	LPL	Cholesterol metabolism, heparin-binding/calcium-binding, Plasma membrane/extracellular space
Q58DP6	Ribonuclease A family member 4	RNASE4	Proteins involved in spermatogenesis and sperm capacitation; have antioxidant function, which protect sperm against the immune system in the female reproductive tract
F1N430	TIMP Metalloproteinase inhibitor 2	TIMP2	Controls the activity of ADAMs (a disintegrin and metalloproteinase), proteins that function in cell adhesion, proteolysis of cell surface components and ECM. ADAMs participate in sperm-egg interactions
P00669	Ribonuclease, RNAse A family, 1 (pancreatic); Seminal ribonuclease	RNASE1; SRN	Sperm capacitation; antioxidant function; catalytic activity; immunosuppression
Q2KJD2	Vesicle-associated membrane protein 3	VAMP3	Vesicle-mediated transport/SNARE complex assembly/protein transport, SNAP receptor activity/syntaxin binding/plasma membrane/cytosol
P81019	Binder of sperm 5; Seminal plasma protein BSP-30 kDa	BSP5	Sperm capacitation/single fertilization/positive regulation of sperm capacitation/phospholipid efflux, *binding* heparin, extracellular space/cell surface
Q3SZT9	Cytochrome c, testis; cytochrome c 2	CYCT	Positive regulation of intrinsic apoptotic signaling pathway, apoptotic process, mitochondrial intermembrane space
Q9BGI1	Peroxiredoxin-5, mitochondrial	PRDX5	Cellular response to ROS or oxidative stress, mithocondrion/nucleus/peroxisomal matrix
Q08DD1	Arylsulfatase A	ARSA	*Binding* sperm to zona pellucida. calcium ion binding, plasma membrane
Q4R0H2	Spermadhesin 2	SPADH2	Single fertilization
P29392	Spermadhesin-1	SPADH1	Single fertilization, extracellular region
**4-year-old group**			
Q3T010	Phosphatidylethanolamine-binding protein 4	PEBP4	Protein phosphorylation signaling cascade; expressed in corpus epididymis
A6QQA8	Quiescin sulfhydryl oxidase 1; Sulfhydryl oxidase	QSOX1	Catalyzes the oxidation of sulfhydryl groups in peptide and protein thiols to disulfides with the reduction of oxygen to hydrogen peroxide
P13696	Phosphatidylethanolamine-binding protein 1	PEBP1	Decapacitation factor (negative regulation of MAPK cascade, ATP binding, cytoplasm
F1N1I6	Gelsolin	GSN	Actin-binding molecule; maintains actin polymerization; regulated by calcium; triggers acrosome reaction
A5D9D2	Complement component 4 binding protein, alpha chain	C4BPA	Binding sperm to zona pellucida
F1N3Q7	Apolipoprotein A4	APOA4	Cholesterol *efflux*/phospholipid efflux/ /cholesterol transfer activity, cell surface
≥**10-year-old group**			
Q2KJE5	Glyceraldehyde-3-phosphate dehydrogenase, testis-specific	GAPDHS	Glycolytic enzyme; essential for generation of ATP; play roles in sperm motility and male fertility; binds to sperm fibrous sheath
A5D792	Deoxycytidine kinase; Histone H4	DCK	Structural constituent of chromatin, DNA binding, nucleus/nucleosome
E1BDA8	Izumo sperm-egg fusion 1	IZUMO1	Sperm-egg recognition/fusion of sperm to egg plasma membrane involved in single fertilization, signaling receptor binding, acrosomal membrane/plasma membrane
Q3MHM5	Tubulin beta-4B chain	TUBB4B	Microtubule-based process, structural constituent of cytoskeleton, microtubule/intercellular bridge/cytoplasm; Major components of sperm microtubules; binds to GTP; involved in the mechanism of sperm motility
F2Z4K0	Tubulin alpha chain	TUBA3E	Microtubule-based process, structural constituent of cytoskeleton, microtubule /cytoplasm
**2 and 4-year-old group**			
P00514	cAMP-dependent protein kinase type I-alpha regulatory subunit	PRKAR1A	cAMP binding, sperm connecting piece/plasma membrane raft/ cytoplasm/axenome
Q9N2I2	Serpin family A member 5; Plasma serine protease inhibitor	SERPINA5	Single fertilization, heparin binding, acrosomal membrane
A6QPE3	Testis expressed 101; TEX101 protein	TEX101	Regulation of flagellated sperm motility/binding of sperm to zona pellucida, plasma membrane raft/plasma membrane) fertilization (single fertilization), sperm motility (flagellated sperm motility),
F1MZX2	Serpin family E member 2; Serine protease inhibitor clade E member 2	SERPINE2	Proteolysis/heparin binding, cytosol/extracellular space
**Four and** ≥**10-year-old group**			
F1N206	Dihydrolipoyl dehydrogenase	DLD	Sperm capacitation/gastrulation, pyruvate dehydrogenase (NAD+) activity/dihydrolipoyl dehydrogenase activity, nucleus/ motile cilium/ mitochondrion/acrosomal matrix
E1B8N5	ATPase Na+/K+ transporting subunit alpha 4; Sodium/potassium-transporting ATPase subunit alpha	ATP1A4	Spermatogenesis/regulation of membrane potential/ flagellated sperm motile/fertilization, ATP binding/ATP hydrolysis activity, sperm midpiece/plasma membrane/membrane raft
F1MY02	Disintegrin and metalloproteinase domain 1b; Disintegrin and metalloproteinase domain-containing protein 1a-like	ADAM1B	*Binding* sperm to zona pellucida/ male gonad development/proteolysis, metalloendopeptidase activity/metal ion binding, plasma membrane
G5E622	ADAM metallopeptidase domain 20	ADAM20	Male gonad development, metalloendopeptidase activity/metal ion binding, sperm head plasma membrane/plasma membrane/external side of plasma membrane

### Gene ontology analysis of the proteins

**Figure 2:**
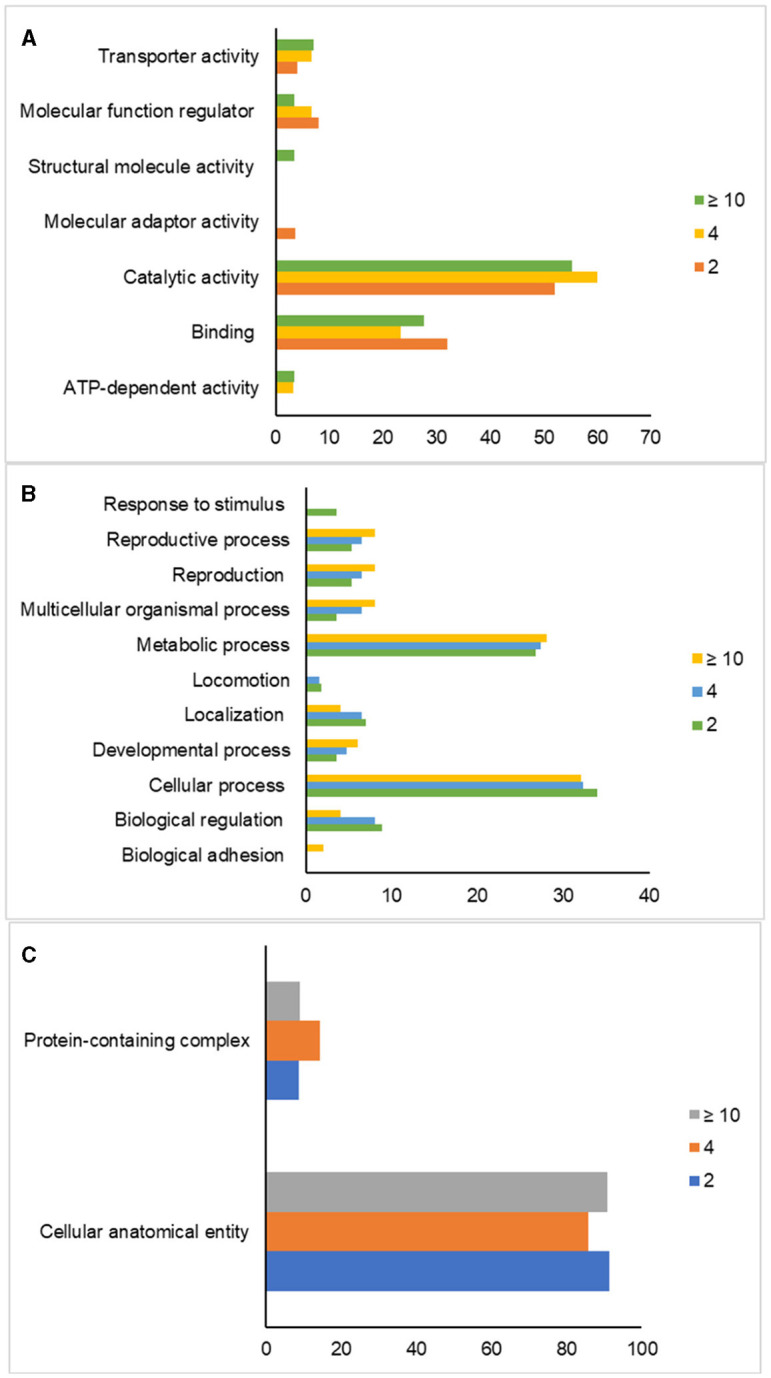
Gene ontology analysis of post-thaw sperm in different ages of bulls. Classification of proteins based on their molecular functions **(A)**, biological processes **(B)**, and cellular components **(C)** in sperm.

Gene ontology analysis classified the proteins based on their biological processes, molecular functions, and cellular components in sperm, as indicated in Figure 2. The most dominant biological processes in sperm across all age groups were cellular (GO:0009987) and metabolic processes (GO:0008152). Response to a stimulus (GO:0050896) was specifically expressed in the 2-year-old group (PRDX5 and TIMP2), while biological adhesion (GO:0022610) was present only in the ≥10-year-old group (IZUMO1). Locomotion, represented by the protein TEX101, was expressed in both 2 and 4-year-old groups. The 2-year-old group expressed proteins TEX101, ZPBP, and SPACA1 related to reproduction (GO:00000003) and reproductive processes (GO:0022414). The 4-year-old group had proteins TEX101, ZPBP, ADAM1B, and ADAM20 associated with reproduction, while the ≥10-year-old group contained ADAM20, ADAM1B, IZUMO1, and ZPBP. The most dominant molecular functions of sperm across all age groups were binding (GO:0005488) and catalytic activity (GO:0003824). The 2-year-old group expressed VAMP3 involved in molecular adaptor activity (GO:0060090), while the ≥10-year-old group had proteins that participated in structural molecule activity, particularly Tubulin (GO:0005198). ATP-dependent activity in the form of the ATP1A4 protein is only expressed in the age group 4 and over 10 years. Furthermore, the most dominant cellular component of sperm in all age groups is the cellular anatomical entity (GO:0110165).

### Post-thaw sperm protein interaction

**Figure 3:**
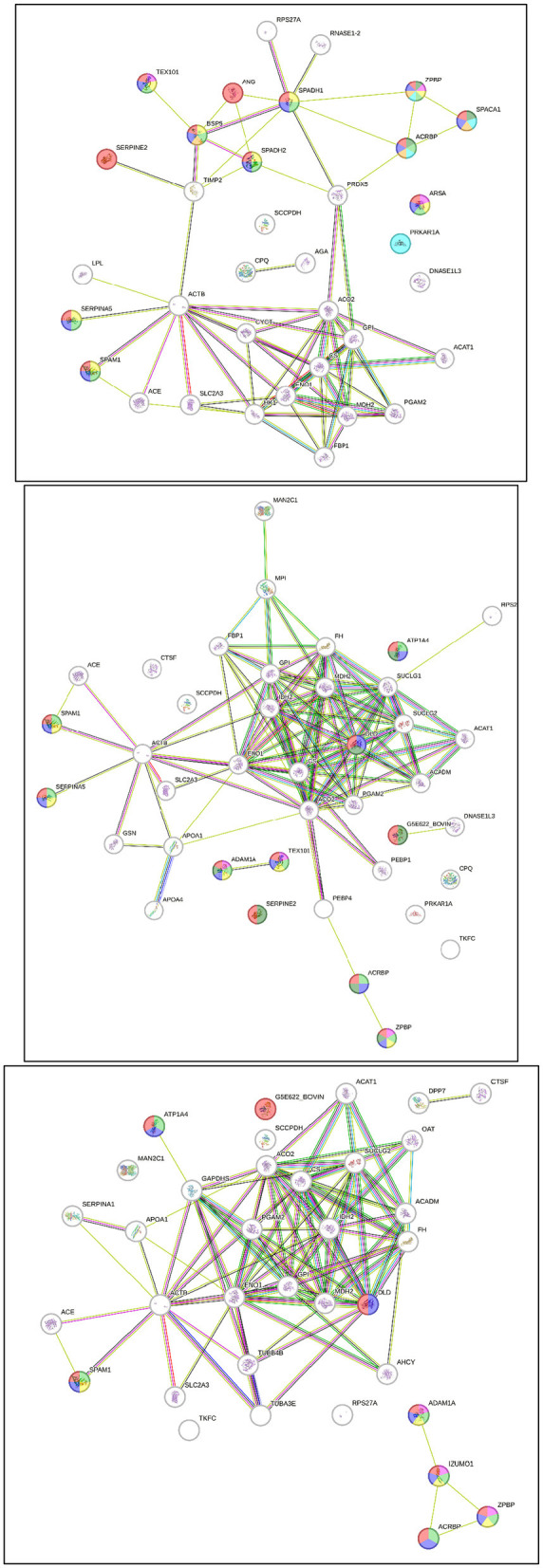
Post-thaw sperm protein interaction at different ages of bull, 2-year-old **(top)**, 4-year-old **(middle)**, ≥ 10-year-old **(bottom)**.

The interactions between post-thaw sperm proteins in different age groups of Simmental bulls were analyzed using STRING, as indicated in Figure 3. Table 2 shows the direct interactions between these proteins related to reproductive processes. Furthermore, the results showed that the sperm proteins found in all age groups are linked to reproductive functions, such as the reproductive process (GO:0022414), sexual reproduction (GO:0019953), fertilization (GO:0009566), and single fertilization (GO: 0007338). Proteins expressed in the 2-year-old group were associated with acrosome assembly (GO:0001675), cellular component assembly involved in morphogenesis (GO:0010927), and spermatid development (GO:0007286). In contrast, those in the -year-old and 2-year-old groups were linked to binding sperm to zona pellucida (GO:0007339). Additionally, ≥10-year-old groups had proteins associated with sperm egg recognition (GO:0035036).

**Table 2:** STRING analysis of post-thawing sperm proteins at different ages of bulls related to the reproductive process.

***GO-term***	**Biological process**	**False discovery rate**	**Protein interaction in the string analysis (the same color)**	**Type of protein**
**A. 2-year-old**			
GO:0022414	Reproductive process	0.00010	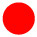	ARSA, ACRBP, SPACA1, ZPBP, SPADH1, SPADH2, BSP5, ANG, TEX101, SERPINE2, SERPINA5, SPAM1
GO:0019953	Sexual reproduction	8.51e-05	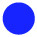	ARSA, ACRBP, SPACA1, ZPBP, SPADH1, SPADH2, BSP5, TEX101, SERPINA5, SPAM1
GO:0009566	Fertilization	5.69e-08	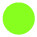	ARSA, ACRBP, ZPBP, SPADH1, SPADH2, BSP5, TEX101, SERPINA5, SPAM1
GO:0007338	Single fertilization	2.10e-07	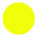	ARSA, ZPBP, SPADH1, SPADH2, BSP5, TEX101, SERPINA5, SPAM1
GO:0007339	Binding sperm to zona pellucida	0.0151	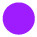	TEX101, ZPBP, ARSA
GO:0001675	Acrosome assembly	0.0031	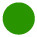	ACRBP, SPACA1, ZPBP
GO:0010927	Cellular component assembly involved in morphogenesis	0.0059	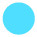	ACRBP, SPACA1, ZPBP, PRKAR1A
GO:0007286	Spermatid development	0.0298	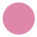	ACRBP, SPACA1, ZPBP, BSP5
**B. 4-year-old**			
GO:0022414	Reproductive process	0.0063	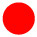	ZPBP, ACRBP, SERPINE2, ADAM1A, TEX101, DLD, ATP1A4, SERPINA5, SPAM1
GO:0019953	Sexual reproduction	0.0060	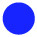	ZPBP, ACRBP, ADAM1A, TEX101, DLD, ATP1A4, SERPINA5, SPAM1
GO:0009566	Fertilization	1.89e-05	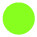	ACRBP, ZPBP, TEX101, ADAM1A, ATP1A4, SERPINA5, SPAM1
GO:0007338	Single fertilization	0.0022	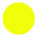	ZPBP, ADAM1A, TEX101, SERPINA5, SPAM1
GO:0007339	Binding sperm to zona pellucida	0.0189	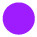	TEX101, ZPBP, ADAM1A
GO:0003006	Developmental process involved in reproduction	0.0426	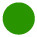	ZPBP, ACRBP, SERPINE2, ADAM1A, DLD, ATP1A4
**C**. ≥**10-year-old**			
GO:0022414	Reproductive process	0.0421	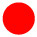	ZPBP, ACRBP, ADAM1A, IZUMO1, DLD, SPAM1, ATP1A4
GO:0019953	Sexual reproduction	0.0119	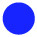	ZPBP, ACRBP, ADAM1A, IZUMO1, DLD, SPAM1, ATP1A4
GO:0009566	Fertilization	0.00015	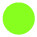	ZPBP, ACRBP, ADAM1A, IZUMO1, SPAM1, ATP1A4
GO:0007338	Single fertilization	0.0144	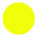	ZPBP, ADAM1A, IZUMO1, SPAM1
GO:0035036	Sperm-egg recognition	0.0183	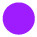	IZUMO1, ZPBP, ADAM1A

## Discussion

In this study, the expression of sperm proteins in different age groups of Simmental bulls and their relevance to sperm functions and fertility were examined. The results showed that proteins associated with essential sperm functions, particularly the metabolic process (GO:0008152), were expressed in all age groups of bulls. The sperm metabolic process is necessary for energy production, which fuels various sperm functions. Bull sperm fulfills its energy requirements through two main metabolic pathways: glycolysis and tricarboxylic acid cycle (28–30). Glycolysis breaks down sugar into pyruvate or lactate substrates, generating energy in the cytosol of sperm (30). These substrates then diffuse into the mitochondria through the pyruvate carrier and become decarboxylated by pyruvate dehydrogenase (PDH) to form acetyl-CoA, a citric acid cycle. The citric acid cycle produces ATP and other adenine derivatives, such as NADH and FADH2, which are converted into ATP through the oxidative phosphorylation pathway in the electron transport chain (30, 31).

Based on the analysis, several sperm proteins were found in all age groups of bulls, such as PGK2 (44.7 kDa; pl 8.32), ENO1 (47.3 kDa; pl 6.8), and GPI (62.8 kDa; pl 7.71), related to glycolysis function (GO:0006096). Furthermore, the analysis results also found sperm proteins such as ACO2 (85.3 kDa; pl 7.83), CS (51.7 kDa, pl 8.12), and MDH2 (35.6 kDa; pl 8.54), related to processes in the tricarboxylic acid cycle (GO:0006099). Previous studies reported these metabolic proteins associated with sperm functions: motility and fertility. Proteins such as PGK2 (32, 33), GPI (34), and ENO1 (16, 35) were involved in sperm motility, while decreased expression of mitochondrial proteins, including ACO2 and MDH2, could impact fertility (1, 16, 36). According to the results, the number of proteins associated with sperm metabolism might vary with age, potentially influencing fertility. This was consistent with previous reports indicating that aging in bulls could lead to mitochondrial dysfunction, affecting metabolic processes (37, 38).

Specifically, a sperm protein related to the process of acrosome assembly and spermatid development, known as SPACA1 (31 kDa; pl 4.85), was found in the 2-year-old group. SPACA1 interacting with ACRBP and ZPBP plays a role in the acrosome assembly function (GO:0001675), and SPACA1 interacting with BSP5 and ACRBP plays a role in the spermatid development function (GO:0007286). The impairment of SPACA1, often synthesized in the testis during spermatogenesis (35, 39), could lead to nuclear plate damage and an abnormal shape of the sperm head (40, 41). The expression of this protein indicated that the 2-year-old age group was still going through the sperm development process.

The 4-year-old group showed specific protein expressions, including PEBP1 (21 kDa; pI 7.49) and PEBP4 (25.4 kDa; pI 6.29). PEBP1 located in the acrosomal cap, post-acrosomal region, and flagella (42) inhibits sperm capacitation or acts as a decapacitation factor by binding to glycosylphosphatidylinositol (GPI)-anchored receptors (13, 22). Inhibiting post-thaw sperm capacitation in the 4-year-old age group permitted energy storage, ensuring the viability and quality of sperm while encountering the oocyte. PEBP4, expressed in the tail of spermatozoa, is associated with sperm motility regulation (43) performed through the Pi3k/Akt signaling cascade and serine/threonine phosphorylation (43, 44). Abundant expression of PEBP1 has been associated with high fertility in bull sperm (13, 22), and PEBP4 is more numerous in fertile bull sperm compared to the infertile counterpart (43).

Both the 2 and 4-year-old age groups expressed TEX101 (27.3 kDa; pI 6.49), which was a GPI-anchored glycoprotein synthesized in testicular germ cells (45) and found in the plasma membrane of spermatocytes, spermatids, and mature sperm (46, 47). TEX101 plays a role in single fertilization (GO:0007338) once interacting with proteins SPADH2, BSP5, SPADH1, ATP1A4, ZPBP, ARSA, SPAM1, and SERPINA5. Furthermore, it is involved in sperm binding to the zona pellucida (GO:0007339) during interaction with ZPBP and ARSA. The binding of TEX101 to cumulus cells induces calcium mobilization and progesterone production, facilitating the acrosome reaction and penetration of the cumulus-oocyte layer (47). The absence of TEX101 expression in older age groups may impact bull fertility since the protein has been validated as a fertility biomarker in mice (46, 47).

The proteins α-tubulin (49.9 kDa; pl 5.1) and β-tubulin (49.8 kDa; pl 4.89) were found to be expressed in the sperm of bulls aged more than 10 years, where these proteins were expressed explicitly in intracellular organelles (GO:0043229). This intracellular organelle, known as the cytoskeleton, plays a vital role in maintaining the morphological integrity of sperm (GO:0005200). These proteins form heterodimers, mainly constituting microtubules (48, 49), which play a role in hyperactive sperm motility during capacitation (48–51). Tubulin expression is associated with structural changes in sperm due to capacitation (48). Furthermore, various mechanisms are involved in the hyperactive movement, such as calcium and cAMP regulation, CATSPER channel functioning, flagella protein phosphorylation, and inhibition of dynein activity (51, 52). A previous study indicated a positive relationship between protein phosphorylation and tubulin distribution along the sperm flagellum during capacitation and acrosome reaction (50). The tubulin expression in post-thaw sperm aged ≥ 10 years suggests high sperm capacitation characterized by hyperactive movement.

## Conclusions

This research shows that the age of bulls influences differences in the expression of the sperm proteome after thawing. The proteomes found in sperm in each age group are related to metabolic processes. Each age group has specific proteins that are expressed, such as SPACA1 and TEX101, which are only found in the 2 and 4-year age groups; PEBP1 and PEBP4, which are only found in the 4-year age group; and tubulin, which is only found in the two age groups over 10 years. Further research is highly expected from the findings of this research, including quantifying the proteomes expressed in the sperm of each age group and carrying out additional studies using *in vivo* and *in vitro* fertility level approaches. Furthermore, although the number of bulls used in this research is a limitation, the results can be an essential reference for further study with a concept similar to that of a more significant number of bulls.

## Data availability statement

The data presented in the study are deposited in the jPOST repository, accession number JPST003244.

## Ethics statement

Ethical approval was not required for the studies on animals in accordance with the local legislation and institutional requirements because only commercially available established cell lines were used.

## Author contributions

FS: Conceptualization, Data curation, Investigation, Methodology, Validation, Visualization, Writing – original draft. NK: Conceptualization, Supervision, Validation, Writing – review & editing. MS: Conceptualization, Supervision, Validation, Writing – review & editing. EK: Conceptualization, Supervision, Writing – review & editing. BPP: Data curation, Formal analysis, Funding acquisition, Project administration, Validation, Visualization, Writing – review & editing. BP: Conceptualization, Formal analysis, Funding acquisition, Project administration, Supervision, Writing – review & editing.
